# Comparative evaluation of the effects of bimaxillary and mandibular setback surgery on pharyngeal airway space and hyoid bone position in skeletal class III patients

**DOI:** 10.4317/jced.59542

**Published:** 2022-05-01

**Authors:** Chaitra Kori, Prajwal Shetty, Mukul Shetty, MS Ravi

**Affiliations:** 1 Orthodontics and Dentofacial Orthopaedics, Private Clinic, Hubli, Karnataka, India; 2 Department of Orthodontics and Dentofacial Orthopaedics, AB Shetty Memorial Institute of Dental Sciences, NITTE (Deemed to be University), Derlakatte, Karnataka, India

## Abstract

**Background:**

To compare the effects of bimaxillary surgery ( Maxillary advancement and mandibular setback) and mandibular setback surgery (Bilateral Sagittal Split Osteotomy) on the pharyngeal airway space (PAS) and the hyoid bone position in a skeletal class III patients.

**Material and Methods:**

Thirty four subjects (21 males, 13 females, mean age 26.5 ± 8 years) with skeletal class III pattern (ANB angle of -2° to -6°) were divided into two groups of equal sizes. Group A consisted of 17 individuals who underwent Bilateral Sagittal Split Osteotomy (BSSO)and Group B consisted of 17 individuals who underwent bimaxillary surgery. In both the group, lateral cephalograms were taken, traced and analyzed for the specified parameters at 3 intervals, pre treatment (C1), post surgical (C2), and post orthodontic treatment (C3). Changes in PAS was evaluated at 3 levels i.e, nasopharynx (Upper PAS), oropharynx (Middle PAS) and hypopharynx (Lower PAS). Changes in hyoid bone position were evaluated in anteroposterior and vertical direction at all the 3 intervals.

**Results:**

There was a significant constriction of airway at oropharyngeal and hypo-pharyngeal level at C2 and C3 in both the groups. However, the reduction at the oropharyngeal airway was greater in group A. In group B, there was significant increase in the airway at the level of nasopharynx, Hyoid bone was positioned more posteriorly post-surgery in group A which did not return to its original position post treatment. In group B hyoid bone was positioned postero-inferiorly post surgically which came back to its original position by the end of orthodontic treatment.

**Conclusions:**

Patients undergoing bimaxillary surgery showed a significant increase in the airway at the level of nasopharynx. Hyoid bone returned to its original position by the end of orthodontic treatment in the bimaxillary surgery group. This study suggested that while treating a skeletal class III malocclusion it is advised to perform maxillary advancements along with mandibular setback surgery.

** Key words:**Bimaxillary surgery, Hyoid bone, Bilateral Sagittal Split Osteotomy, Pharyngeal airway space.

## Introduction

Orthognathic surgery involves repositioning of maxilla and/or mandible in patients with severe skeletal discrepancy in whom dentofacial discrepancy cannot be corrected by orthodontic camouflage and/or by growth modification therapy due to completion of growth. The frequent reason for undergoing such surgery is to achieve esthetic enhancement of the dentofacial complex with better masticatory function. However, surgery is not a substitute for orthodontic treatment. Rather, it must be properly coordinated with orthodontics to achieve better and stable results (1). One such entity which require surgical approach is skeletal class III deformity.

Class III conditions are usually a result of anteroposterior discrepancies which may consist of either a true mandibular prognathism or maxillary deficiency or combination of both. Earlier correction of such class III sagittal discrepancy had been achieved by isolated mandibular setback surgeries. Later studies reported, only 20-25% of class III individuals presents with true mandibular prognathism, whereas other 75% of individuals will have sagittal maxillary deficiency associated with it (2). Thus, with the advancement in knowledge and techniques, surgical approach for such cases progressed to bi-maxillary surgery.

Surgical repositioning of bony facial skeleton will inevitably affect soft and hard tissue relationship. Surgical repositioning of the maxillo-mandibular complex results in varying changes in the volume and area of the nasal and oral cavities (3,4). The upper airway is of more concern for medical professionals who are dealing with maxillofacial region. It is very crucial to know about airway due to its association with craniofacial development and respiratory disorders. However, this depends on the amount of correction and its direction. Subsequently, the changes may influence the treated patient’s quality of sleep in long term (5).

Mattos *et al*. (6) reported that, during surgical correction of skeletal class III deformity there is a significant decrease in the oropharyngeal airway in mandibular setback surgery, whereas in bimaxillary surgery there is a milder decrease and an increase in airway after maxillomandibular advancement surgery.

Former studies have reported isolated mandibular setback surgeries bring about alterations in the tongue and hyoid bone position, as well as the size of the pharyngeal airway (7-10). Following surgery, the hyoid bone generally is seen to displace postero-inferiorly, bringing the tongue into a postero-inferior position as well (11). This alteration in position of tongue is shown to cause a reduction in the pharyngeal airway space (12).

There are conflicts of opinions regarding the duration and extent of post-operative alterations in the oropharyngeal complex, particularly about pharyngeal airway dimension and position of hyoid bone. Few authors have showed that, only short term alterations were observed following mandibular setback surgery which did not sustain for long time post-surgery (9-11). Enacar *et al*. (3) and kawakami *et al*. (10) reported that, hyoid bone assumed its original position post- surgery. However, others like Guven *et al*. (11), tselnik *et al*. (13) reported that hyoid bone never came back to its preoperative position following surgery.

Postoperative alterations in pharyngeal complex may compromise the skeletal stability and the airway size postoperatively. The positional changes of hyoid bone are determined by the combined action of supra and infra hyoid muscles and the resistance provided by the elastic membranes of trachea and larynx (14,15). The postoperative alteration in the position of hyoid bone may bring about relaxation of the suprahyoid muscles, which in turn will alter the head and neck muscle balance, causing forces directed more anteriorly by the muscles of neck, resulting in forward pull of the mandible (11). If this influence continue for prolonged period, it might result in skeletal relapse.

The change in the pharyngeal airway space following mandibular setback surgery is a risk factor for developing obstructive sleep apnea (OSA). OSA is thought to be a risk factor for systemic and pulmonary hypertension and cardiac arrhythmias. Thus, in recent era the frequency of performing isolated BSSO has gone down to 10% and bi-jaw surgery has gone up to 40% in skeletal class III deformities (16).

Many studies have investigated the effect of orthognathic surgery on pharyngeal airway space in skeletal class III deformity cases. however most of them studied the effect of mandibular setback surgery alone. Thus, the aim of this study is to compare the effects of bimaxillary and BSSO on PAS and hyoid bone position in skeletal class III deformities.

## Material and Methods

-Subjects

In this retrospective study, 34 adult patients (21 males & 13 female, mean age 26.5 ± 8 years) who underwent either isolated mandibular setback surgery or bimaxillary surgery for the correction of skeletal class III deformity were selected for the study, after getting an approval from the institutional Review Board (IRB) and Ethical Committee ( ABSM/EC47//2017). Patients with a skeletal class III pattern with ANB angle of -2° to -6° and no history of previous orthodontic treatment were divided into two groups. The first group consisted of 17 individuals ( 10 male and 6 female) who were treated with isolated bilateral sagittal split osteotomy and the second group comprised of 17 individuals ( 11 male and 7 female) were treated by lefort I with bilateral sagittal split osteotomy.

-Inclusions and Exclusion Criteria

Patients presenting with skeletal class III deformity with mandibular prognathism, within the age group of 18 – 35 years, to ensure growth completion were included in the study. Only those individuals who underwent BSSO (Group A) or bimaxillary surgery (Group B) along with presurgical and postsurgical orthodontic treatment in order to create a sTable postoperative occlusion were considered. Individuals with habitual snoring, obstructive sleep apnoea(OSA), chronic airway diseases or with previous history of tonsillectomy or adenoidectomy were excluded from the study.

**Figure 1:**
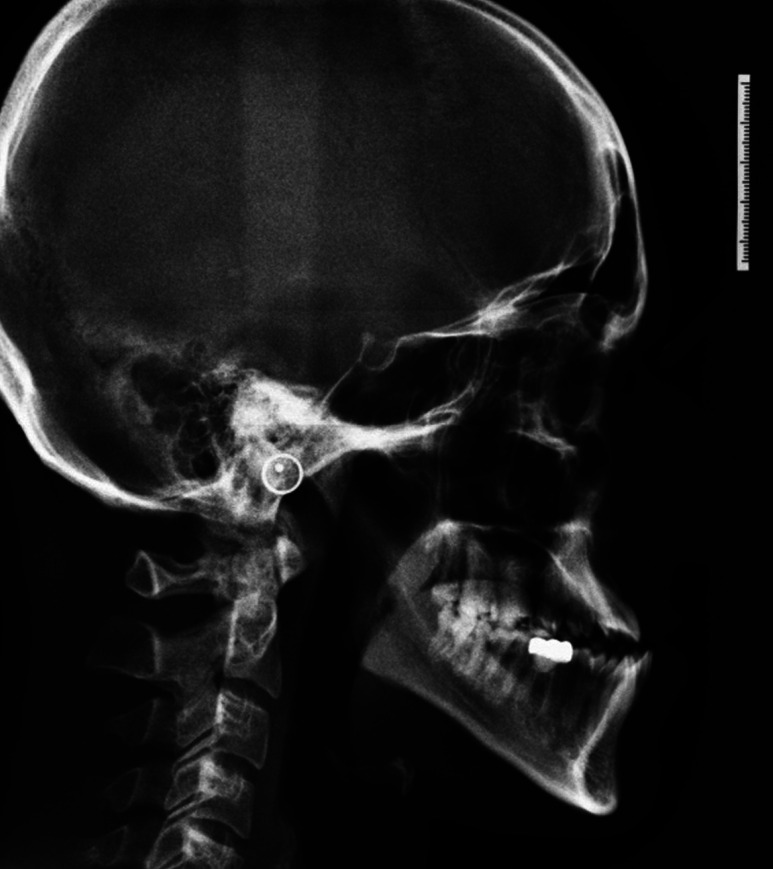
Pre-orthodontic lateral cephalogram.

**Figure 2:**
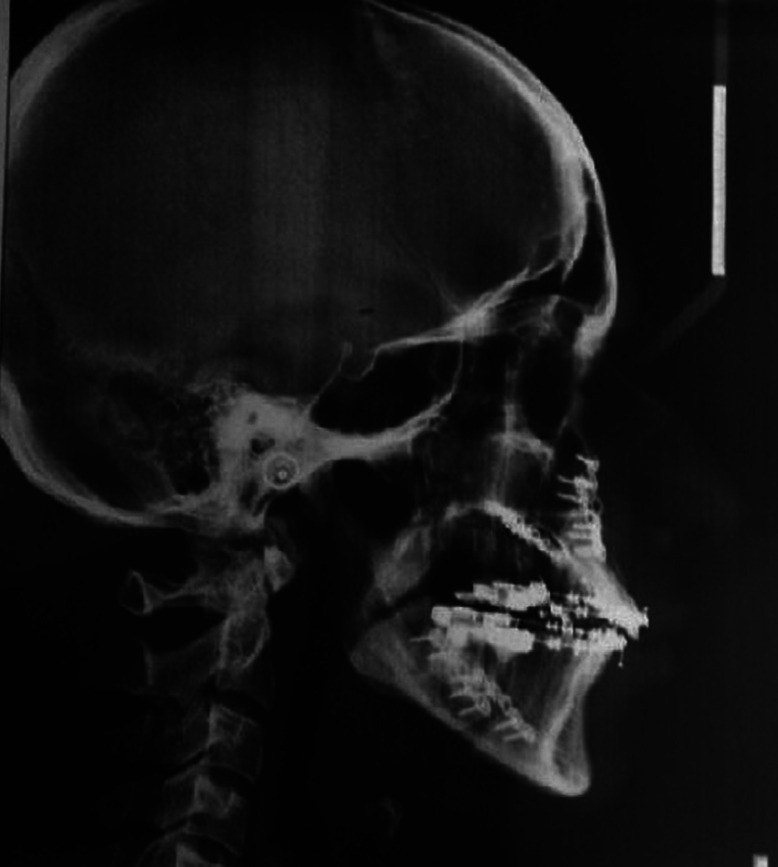
Post-surgical lateral cephalogram.

**Figure 3:**
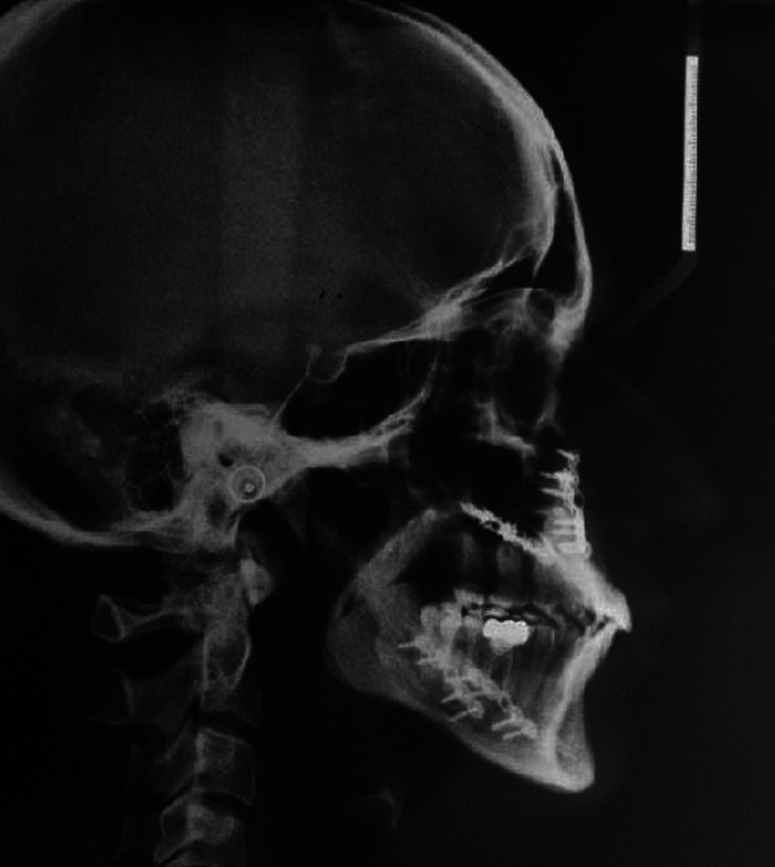
Post-orthodontic lateral cephalogram.

-Preparation of sample:

Lateral cephalograms of 34 individuals who had reported to the department of orthodontics for the treatment of class III malocclusions were selected from the archive. In both the group, lateral cephalograms were taken at 3 intervals, pre treatment (C1) (Fig. 1), post surgical (C2) (Fig. 2), and post orthodontic treatment (C3) (Fig. 3). Radiographs of all patients were taken in the upright position with the Frankfort horizontal line parallel to the floor, tongue and lips in relaxed position, and jaws in habitual occlusion.

**Figure 4:**
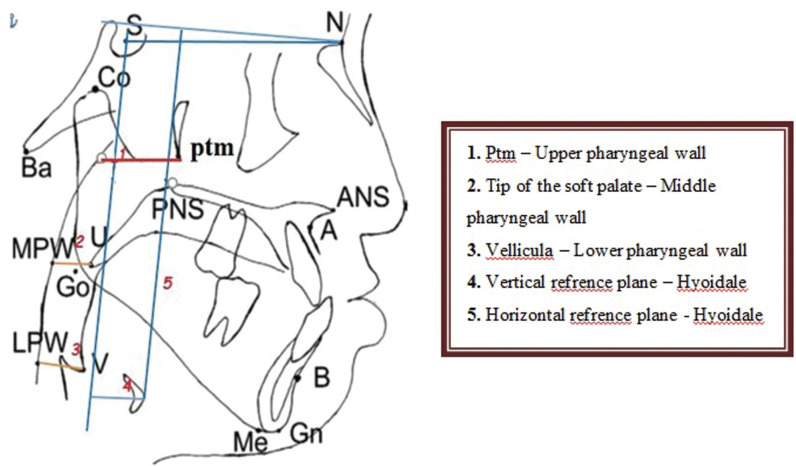
CEPHALOMETRIC LANDMARKS: Sella (S): The point representing the midpoint of the pituitary fossa or Sella turcica.Nasion (N): The most anterior point of frontonasal suture in the mid- sagittal plane. Ptm point (ptm): It is a intersection of the inferior border of foramen rotundum with posterior wall of pterygo-maxillary fissure. Posterior pharyngeal wall (PPW): the radiographic outline of the posterior wall of pharynx.U point: The tip of the soft palate. Vallicula (V pt): the tip of the epiglottis is vallicula. The deepest point of vallicula is considered. Hyoid (Hy): The most superior and anterior point on the body of hyoid.

-Measurements:

All cephalograms are taken with planmeca promax (planmeca Oy, Finland) with the patients head in natural head position (mirror position). The cathode to object distance was standardized at 60 inches or 5 feet, at 5mA and 68 kVp, with an exposure time of 18.7 seconds. All cephalograms were traced with a 0.5 mm HB lead pencil on a 26 micron matte acetate paper. Landmarks used in the study are shown in the Figure 4. The data obtained were analysed and compared to obtain results.

Parameters for assessing PAS (Ptm – UPW, V – LPW, U - MPW) was determined according to a previous study (15,16). The pharyngeal airway size was evaluated using lateral cephalograms . Although lateral cephalogram is a 2 dimensional image of pharyngeal airway, it has been used extensively in assessing the craniofacial form and pharyngeal airway space. Studies have showed that, pharyngeal airway space measured by cephalograms was highly correlated with measurements obtained with three dimensional computed tomography scan with the high accuracy in predictability (17,18). Due to the addition benefits like, less radiation exposure and cost effectiveness of lateral cephalograms it was chosen for measuring the PAS changes at three levels.

Changes in PAS was evaluated at 3 levels i.e, nasopharynx, oropharynx and hypopharynx. Changes in hyoid bone position were evaluated in anteroposterior and vertical direction at all the three intervals.

-Statistical analysis:

Collected data was analyzed using Unpaired t test between 2 groups. Repeated measures ANOVA test was used to find differences in the pharyngeal airway space at 3 different level in both groups. Bonferroni Pairwise comparison showed significant changes in the airway space on comparing C1 with that of C2 and C3 in both the groups. Repeated measures ANOVA test compared changes in anteroposterior and vertical positioning of hyoid bone at 3 intervals in both group. *P*<0.05 was considered significant. All the data collected were statistically analyzed using the statistical software SPSS (version 20.0 Armonk, NY: IBM Corp).

## Results

The mean value and standard deviation for the pharyngeal airway space and hyoid bone position were calculated in bimaxillary surgery group and BSSO group.

-Comparison within BSSO:

The Repeated measures ANOVA test comparing the difference within the BSSO ( Table 1) showed that

**Table 1:**
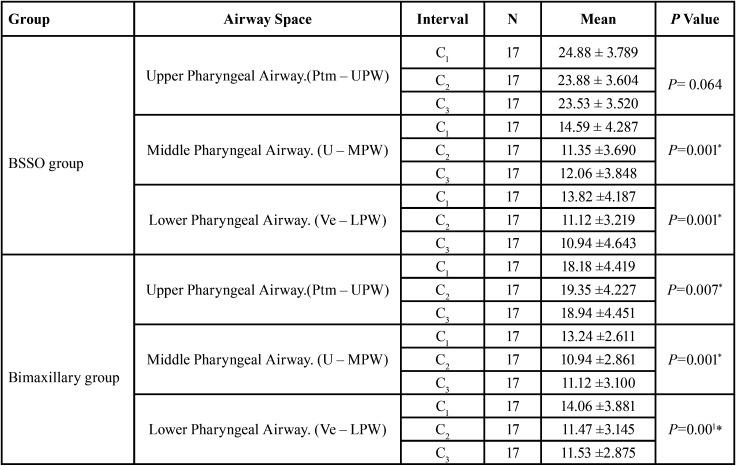
Comparison of pharyngeal airway space in BSSO group and Bimaxillary group. P value ≤ .05 is considered significant.

The changes in the upper pharyngeal airway (Ptm – UPW) from pre orthodontic to post-surgical to post orthodontic i.e, from C1 to C2 to C3 had no significant difference with the mean difference of 24.88 ± 3.78, 23.88 ± 3.6 and 23.53 ± 3.52 mm respectively.

When the changes in mid pharyngeal airway (U – MPW) was compared, there was a significant reduction in the pharyngeal airway space when compared to C2 and C3 with that of C1. Bonferroni Pairwise Comparisons showed that, on comparing C1 with that of C2 and C3, there was reduction in mid pharyngeal airway with the mean difference of 3.2 and 2.5 with *p* value of 0.010 and 0.017 respectively. However, the changes were non- significant from C2 to C3 (Table 2).

When the changes in lower pharyngeal airway(Ve – MPW) was compared within the BSSO group between C1 to C2 to C, there was a significant reduction in the pharyngeal airway space when compared C2 and C3 with that of pre-orthodontic values. Bonferroni Pairwise Comparisons showed that, on comparing C1 with that of C2 and C3, there was statistically significant reduction in lower pharyngeal airway with the mean difference of 2.7 and 2.8 and *p* value of 0.009 and 0.005 respectively. However the changes were non-significant from C2 to C3 ( Table 2).

**Table 2:**
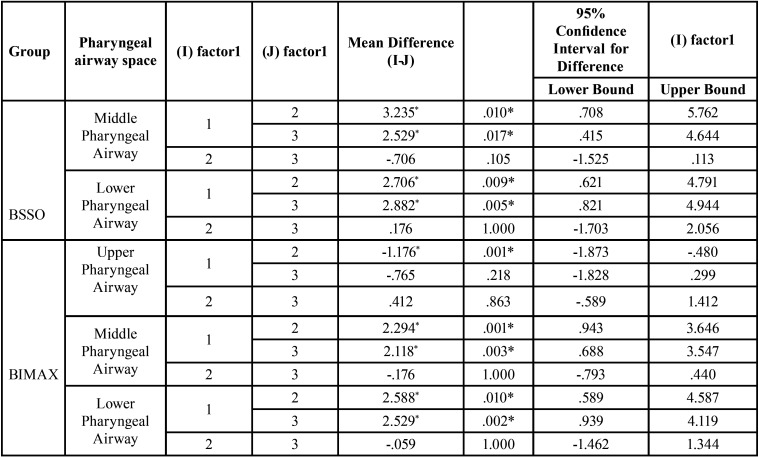
Bonferroni.Pairwise Comparisons between BSSO and Bimaxillary group. P value ≤ .05 is considered significant.

-Changes in hyoid bone position in BSSO:

The results showed that anteroposterior distance of the hyoid bone calculated as the change in length between vertical reference line to hyoidale point, showed a significant change in the position with the pre surgical distance of 6.35 ± 4.4mm, post-surgical distance, and post treatment was 4.53 ± 4.2 and 5.29±4.2 respectively ( Table 3). Pairwise Comparisons of C1 with C2 and C3 showed that the hyoid position changed with the mean difference of 1.82 and 1.05 mm and *p* value of 0.01 and 0.02 respectively. There was no significant change in horizontal positioning of hyoid bone when compared from C2 to C3 ( Table 4).

**Table 3:**
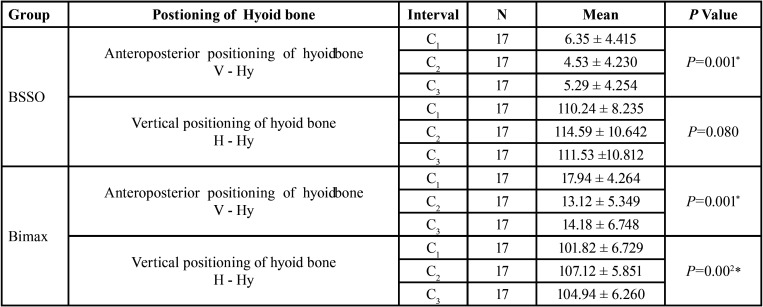
Comparison within BSSO group and Bimaxillary group for anteroposterior positioning of hyoid bone. *P* value ≤ .05 is considered significant.

**Table 4:**
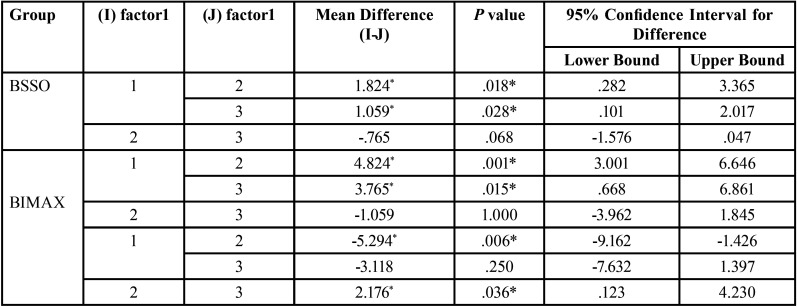
Pairwise Comparisons between BSSO group and bimaxillary group. *P* value ≤ .05 is considered significant.

-Comparison within BSSO group for vertical positioning of hyoid bone

 When the pre treatment vertical distance of the hyoid bone calculated as the change in length between horizontal reference line to hyoidale point had a mean distance of 110.24±8.2 mm, post-surgical distance, and post treatment is 114.5±10 and 111±10.8 respectively ( Table 3). There was no significant change in vertical hyoid positioning from C1 to C2 to C3 .

-Comparison within bimaxillary surgery:

The Repeated measures ANOVA test comparing the difference within the bimaxillary surgery (lefort I with BSSO) group ( Table 1) showed that, 

The changes in the upper pharyngeal airway (ptm – upw) from C1 to C2 to C3 was significant with the mean difference of 18.18 ± 4, 19.35 ± 4 mm and 18.94 ± 4 respectively. Bonferroni.Pairwise Comparisons showed that, on comparing C1 with that of C2, there was statistically significant increase in upper pharyngeal airway with the mean difference of -1.176 mm, *p* value of 0.001. However, the changes were non-significant from C2 to C3 and C1 to C3 ( Table 2).

When the changes in middle pharyngeal airway (U – MPW) was compared within the bimaxillary surgery group between C1 to C2 to C3, there was significant reduction in the PAS from C1 to C2 to C3. Bonferroni Pairwise Comparisons showed that, on comparing C1 with that of C2 and C3, there was statistically significant reduction in mid pharyngeal airway with the mean difference of 2.294 and 2.118 and *p* value of 0.001 and 0.003 respectively. However, the changes were non-significant from C2 to C3 ( Table 2).

When the changes in lower pharyngeal airway(Ve – MPW) was compared within the bimaxillary surgery group between C1 to C2 to C3, there was reduction in PAS (Table 1). Bonferroni. Pairwise Comparisons showed that, on comparing C1 with that of C2 and C3, there was statistically significant reduction in lower pharyngeal airway with the mean difference of 2.58 and 2.52 and *p* value of 0.010 and 0.002 respectively. However the changes were non-significant from C2 to C3 ( Table 2).

-Changes in hyoid bone position in bimaxillary surgery in anteroposterior postioning

The results showed that, the pre orthodontic anteroposterior distance of the hyoid bone in bimaxillary surgery is calculated as the change in length between vertical reference line to hyoidale point had a mean distance of 17.9± 4.2 , C2 and C3 values were 13.12± 5.3 was 14.18±7.8 ( Table 3). Pairwise Comparisons of C1 with C2 and C3 showed that the hyoid position changed with the mean difference of 4.82 and 3.76 mm and *p* value of 0.001 and 0.015 respectively. There was no significant change in horizontal positioning of hyoid bone when compared from C2 to C3 ( Table 4).

-Comparison within Bimaxillary group for vertical positioning of hyoid bone

When the pre treatment vertical distance of the hyoid bone calculated as the change in length between horizontal reference line to hyoidale point had a mean distance of 101.82±6.7 mm, C2 and C3 values were 107.12±5.8 and 104.94±6.2 ( Table 3). Pairwise Comparisons showed that there was significant change in vertical hyoid positioning from C1 to C2 with the mean distance of -5.29 and from C2 to C3 with the mean distance of 2.176 and *P* value of .006 and .036 respectively. However there was no statistically significant difference in distance of hyoid bone from C1 to C3 with the mean distance of -3.118 and *p* value of 0.250 ( Table 4).

## Discussion

The mandible, hyoid bone, base of the tongue, pharyngeal walls are all closely related to each other though their muscular and ligamentous attachments. Since the attachment of genioglossus muscle relates the base of the tongue to the mandible, when the mandible is positioned back there will be relative backward positioning of tongue. Further, the tongue is related to the positioning of the hyoid bone through muscles and connective tissues. Thus any retraction of mandible will lead to posterior positioning of tongue and narrowing of PAS (19).

Decrease in the size of PAS is also attributed to the inflammation and swelling of the soft tissues following the surgery. There will be encroachment of inflamed tissue on the PAS thereby potentially compromising its potency, and this could also be the possible explanation for change in position of hyoid bone as an adaptation to preserve the airway potency. Considering these could be the short term which might return back to their original position once the inflammation subsides. Thus three time periods were considered in this study. Pre-treatment, immediate postsurgical and post orthodontic treatment.

Studies have reported significant increase in the upper pharyngeal airway space which was retained after 8 months following bimaxillary surgery (17,20). The results of the present study also showed increase in upper pharyngeal airway with the mean difference of -1.176 mm, *p* value of 0.001. There was no difference in upper airway size when compared pre surgical and post treatment period(C1 and C3). A possible explanation could be the advancement of velum and velopharyngeal muscle due to maxillary advancement which compensated the constricted effect of BSSO (9) or because the amount of setback of mandible was more in only BSSO group then bimaxillary surgery group. However, in present study there was no significant difference in upper airway space in BSSO group at any interval of time.

The results obtained from this study showed that there were no significant differences seen in the measurements obtained from C2 to C3; whereas, there were significant differences seen from C1 to C2 and from C1 to C3. Indicating that the post-surgical measurements remained stable after 6 months. These results are in agreement with various other studies dealing with the stability of orthognathic surgeries in correcting skeletal class III discrepancies (20,21).

In BSSO surgery, Achilleos *et al*. (22) reported no reduction in oro-pharyngeal or hypopharyngeal sagittal airway dimension, when observed for long term and the reason was due to the compensatory functional readjustments of hyoid bone and surrounding musculature in order to maintain the airway patency in a surgically altered environment.

Whereas Eggenesperger *et al*. (23) reported that the lower pharyngeal airway remained constant but, size of naso and oro pharyngeal airway reduced with time continuosly. Similarly, kawakami *et al*. (10) reported that mandibular setback surgery causes airway narrowing late after surgery, while the early postoperative airway dimension was maintained. However in this study there was significant reduction in the oropharyngeal airway space by 3.2mm from C1 to C2. the possible reason could be soft palate was placed more back due to its continuous contact with the dorsum of the tongue following mandibular setback. Whereas, there was no significant difference in airway dimension from C2 to C3.

The studies have shown reduction in the oro and hypo pharyngeal airway space following bimaxillary surgery but, the reduction was comparatively lesser then that of isolated mandibular setback surgery (17,22,24). The results of this study is in accordance with these studies there was significant reduction of the pharyngeal airway from C1 to C2 by 2.2 mm which was comparatively lesser then in BSSO group in which the reduction was 3.2mm. the possible explanation would be because the maxillary advancement has compensated the pressure of tongue on soft palate. However, there was no significant difference in size from C2 to C3.

Samman *et al*. (9) cakarne *et al*. (25) reported that, there was significant decrease in the airway dimension at the lower pharyngeal airway levels in both mandibular setback and bimaxillary surgery. Several other studies also reported reduction in hypo pharyngeal airway continuously for 1-3years after the setback of mandible (4,8,16). However, in the present study there was statistically significant airway reduction in both groups which was 2.7mm in mandibular setback and 2.6mm in bimaxillary surgery. The possible explanation will be due to the posterior positioning of the tongue with mandible. There was no significant differences in the dimensions from C2 to C3.

Various studies have reported that there was posterior positioning of hyoid bone with mandibular setback surgery (5,7,26,27). Gu *et al*. (28) in a study reported that the tension and length of the supra and infra hyoid muscles was increased postoperatively in the time period of 3 year and that, there was a close relation between suprahyoid muscles and skeletal relapse due to the force created by the muscles to return back to its original resting tension. In contrast, Eggennspeger *et al*. (24) reported that the progressive improvement in the length of the muscle from the immediate post-surgical stage to 12 years postoperatively was associated with the alteration in the position of hyoid bone rather than to the skeletal changes of mandible. Thus concluded that length of the suprahyoid muscle defines its position and it does not contribute to the skeletal relapse.

In present study, when anteroposterior displacement of hyoid bone was considered,the hyoid bone assumed more posterior position in both groups. The possible explanation could be short span of observation. There was inferior positioning of the hyoid bone post operatively in mandibular setback group but the difference was not significant. And there was no difference from C2 to C3. On the other side, in bimaxillary group there was significant inferior positioning of hyoid bone. From C1 to C2. And the hyoid bone was seen to return back to its original position by the end of post-surgical orthodontic treatment.

Reduction in pharyngeal airway space is been reported to be the risk factor for development of obstructive sleep apnea (20). Backward displacement of the base of the tongue were the characters of OSA patients. PAS narrowing is possibly a predisposing factor for OSA. However, in the present study no one have reported snoring or obstructive sleep apnea from either groups.

## Conclusions

After evaluation and comparison of alterations in PAS following Mandibular setback surgery with bimaxillary surgery, It was concluded that, there was increased naso pharyngeal airway in bimaxillary group. Reduction in oropharyngeal airway was seen in both groups but, the reduction was more in BSSO group when compared to that of bimaxillary group. Hypopharyngeal airway was also reduced in both group but there was no significant difference between groups. Hyoid bone was place more inferiorly and posteriorly in bimaxillary group but it tend to return back by the end of the orthodontic treatment. Thus, whenever possible bimaxillary surgery should be preferred over mandibular setback surgery.
